# Serologic evidence of crimean-congo hemorrhagic fever virus exposure among livestock and farmers in the Democratic Republic of the Congo

**DOI:** 10.1371/journal.pntd.0013648

**Published:** 2025-10-24

**Authors:** Megan Halbrook, Boniface Lombe Pongombo, Sydney Merritt, Emmanuel Hasivirwe Vakaniaki, Daddy Kanonge Lubunda, Charlotte Tshingula, Yannick Munyeku-Bazitama, Masahiro Kajihara, Sheila Makiala-Mandanda, Ryan Harrigan, Patrick Mukadi-Kakoni, Nicole A. Hoff, Steve Ahuka Mundeke, Ayato Takada, Augustin Tshibwabwa Twabela, Placide Mbala-Kingebeni, Lisa E. Hensley, Anne W. Rimoin

**Affiliations:** 1 Department of Epidemiology, Jonathan and Karin Fielding School of Public Health, University of California, Los Angeles, California, United States of America; 2 Laboratoire Vétérinaire Centrale de Kinshasa, Kinshasa, Democratic Republic of the Congo; 3 Faculté de Médecine Vétérinaire, Université Pédagogique Nationale, Kinshasa, Democratic Republic of the Congo; 4 Institut National de Recherche Biomédicale, Kinshasa, Democratic Republic of the Congo; 5 Division of Global Epidemiology, International Institute for Zoonosis Control, Hokkaido University, Sapporo, Japan; 6 Division of International Research Promotion, International Institute for Zoonosis Control, Hokkaido University, Sapporo, Japan; 7 One Health Research Center, Hokkaido University, Sapporo, Japan; 8 Department of Medical Biology, University of Kinshasa, Democratic Republic of the Congo; 9 Institute of the Environment and Sustainability, University of California, Los Angeles, California, United States of America; 10 Zoonotic and Emerging Disease Research Unit, National Bio and Agro-Defense Facility, USDA Agricultural Research Service (ARS), Manhattan, Kansas, United States of America; Public Health Agency of Canada, CANADA

## Abstract

**Background:**

Crimean-Congo Hemorrhagic Fever (CCHF) is a potential high-threat zoonotic disease caused by the Crimean-Congo hemorrhagic fever virus (CCHFV). Transmission of CCHFV occurs primarily through bites of infected *Hyalomma* ticks or direct contact with infected animals or humans. This study presents a cross-sectional assessment of CCHFV seroprevalence and risk factors associated with occupational and environmental exposures among cattle, swine, and agricultural workers.

**Methods:**

Nine provinces across the Democratic Republic of the Congo (DRC) were selected and collection took place from June 2023 to July 2024. Five herds per species in each province were randomly visited, and at each facility or herd, up to 20 animals were chosen for serum sampling and attached tick collection. In five provinces, farm workers present on the day of collection were enrolled. Detection of anti-CCHFV Immunoglobulin G (IgG) antibodies was assessed via an in-house nucleoprotein-based enzyme-linked immunosorbent assay (ELISA).

**Results:**

Among the 1,118 cattle surveyed across nine provinces 57.0% (95%CI: 54.1-59.9%) were seroreactive. Cattle from two provinces in the southeast, Tanganyika and Lualaba, had 94.6% (95%CI: 89.9-99.2%) and 90.7% (95%CI: 84.9-96.5%) reactivity, respectively. Among the 1,020 swine surveyed 13.4% (95%CI: 11.1-15.2%) were seroreactive. Among the 180 agricultural workers surveyed, 12.8% (95%CI: 7.9-17.6%) were seroreactive for CCHF antibodies.

**Conclusions:**

This serologic survey indicated that CCHFV is circulating in the DRC and the southeast provinces are particularly at risk for spillover and morbidity among humans. Though no human cases have been reported since 2008, surveillance for CCHF should be considered among veterinary professional and healthcare workers.

## Author summary

This study investigated exposure to Crimean-Congo Hemorrhagic Fever virus (CCHFV), a tick-borne disease, in livestock and agricultural workers across nine provinces of the Democratic Republic of the Congo (DRC). Researchers tested over 1,100 cattle, 1,000 swine, and 180 farm workers for antibodies to CCHFV, which can indicate past infection. More than half of the cattle (57%) and 13% of the swine had evidence of exposure, with extremely high rates in the southeastern provinces of Tanganyika and Lualaba, where over 90% of cattle tested positive. About 13% of farm workers also showed signs of past infection. The findings reveal widespread circulation of CCHFV among animals and people, even though no human outbreaks have been reported in the DRC since 2008. The study also found limited use of protective gear and close daily contact between workers and livestock, highlighting the risk of future spillover events. These results underscore the need for improved surveillance, farmer education, and One Health strategies that integrate human, animal, and environmental health efforts to prevent emerging zoonotic threats.

## Introduction

Crimean-Congo Hemorrhagic Fever (CCHF) is a potential high-threat zoonotic disease caused by the Crimean-Congo hemorrhagic fever virus (CCHFV), a tick-borne virus belonging to the Nairoviridae family, genus *Orthonairovirus* [1,2]. The disease is characterized by the abrupt onset of high fever, headache, myalgia, gastrointestinal symptoms, and in severe cases, hemorrhagic manifestations, multi-organ failure, and death. With a case fatality rate estimated between 10% and 40%, CCHFV is among the most virulent of hemorrhagic fevers affecting humans [3,4]. The World Health Organization (WHO) has designated CCHFV as a priority pathogen and a target of global epidemic preparedness initiatives due to its growing geographical distribution, severe clinical outcomes in humans, potential for nosocomial outbreaks, and lack of mitigation strategies [5,6].

Transmission of CCHFV occurs primarily through bites of infected *Hyalomma* ticks or via direct contact with the blood or tissues of infected animals or humans [7]. Livestock, such as cattle, goats, and sheep can be infected and while livestock mainly experience asymptomatic infection, they are key species in the transmission cycle of the virus to other hosts and humans [8]. Populations at highest risk for disease transmission include farmers, herders, veterinarians, abattoir workers, and butchers, who may encounter infectious materials from animals or humans, or tick infestations through routine animal husbandry or slaughter activities [9,10]. This occupational risk is exacerbated in low- and middle-income countries, where protective equipment, training, and veterinary oversight are often limited.

The Democratic Republic of the Congo (DRC), the second-largest country in Africa in terms of land area, encompassing vast ecological zones that are favorable for maintaining both tick populations and supporting livestock production. Agriculture and animal husbandry comprise a significant portion of the country’s informal economy and livelihood base, particularly in rural areas [11]. The DRC has a fragile public health infrastructure, limited veterinary surveillance, and lack of routine diagnostic capacity that has hampered efforts to monitor or quantify zoonotic diseases such as CCHF. Since the virus was first described and documented in Crimea in the 1940s and first virus isolation in DRC in the 1950s, epidemiological data regarding the scope of CCHF disease risk in central Africa remain sparse and fragmented [12]. To date, there has been no comprehensive One Health assessment of CCHFV prevalence among Congolese livestock and persons who work in close contact with livestock.

This study presents a cross-sectional assessment of CCHFV seroprevalence and risk factors associated with occupational and environmental exposures among livestock (cattle and swine), persons with occupational exposure to livestock including farmers, herders, and abattoir workers. This is among the first integrated One Health studies of CCHFV prevalence in the DRC which links animal and farmer serosurveillance with farm management practices and vector distribution. The results from this study could be used to inform both national policy and international preparedness frameworks. Moreover, they may help to close a critical knowledge gap in an under-studied region of sub-Saharan Africa and to illustrate how strategic global health engagement aligns with national security objectives. The proactive detection of zoonotic viruses, such as CCHFV, at their source contributes not only to the protection of vulnerable populations in endemic areas but also to safeguarding the health and stability of nations far beyond their borders.

## Methods

### Ethics statement

Ethics approvals for this work were provided by Institutional Review Boards of the University of California, Los Angeles (IRB#23-000676) and the Kinshasa School of Public Health, University of Kinshasa, DRC (ESP/CE/161/2024). Animal research ethical review was provided by of the University of California, Los Angeles (ARC#2023-009).

Nine provinces across the DRC were selected based on livestock density reports provided by the Central Veterinary Laboratory in Kinshasa and logistical feasibility: Tanganyika, Lualaba, Kasai, Tshopo, Kasai Oriental, Maniema, Kinshasa, Equateur, and Sud Ubangi. Collection took place from June 2023 to July 2024. Five herds per species in each province were randomly visited, and at each facility or herd, up to 20 animals were chosen for serum sampling and attached tick collection. A brief health survey was conducted for each animal which assessed body condition score, tick load, the presence of lesions, and current illness state, if any. A facility survey was administered to the farm owner or manager to assess the facility management practices that may impact CCHFV transmission such as grazing habits, availability of any veterinary medicines, history of infectious disease among the herd, and others. Up to 20 ticks found on each animal were collected. The dorsal and ventral sides of collected ticks were imaged and speciated using deep-learning algorithms via the IDX machine (Vectech, Baltimore, MD, USA) and confirmed by trained lab staff. In five provinces (Kasai Oriental, Kinshasa, Lualaba, Maniema, Sud Ubangi), all personnel present at the facility were invited to enroll in the study and a serum sample and survey on demographics and occupational and personal risks was collected.

Detection of anti-CCHFV Immunoglobulin G (IgG) antibodies was assessed via an in-house enzyme-linked immunosorbent assay (ELISA) that has previously been described [13]. All sera were heat-inactivated at 56 °C for 30 minutes before testing. The assay involved coating plates with purified CCHFV NP antigen and blocking with 3% skim milk in phosphate-buffered saline (PBS). Serum samples, diluted 1:100 in PBS with 1% skim milk, were then added. Bound antibodies were detected using a horseradish peroxidase–conjugated protein A/G followed by the addition of 3,3’,5,5’-tetramethylbenzidine (TMB) substrate. The reaction was stopped with 1 N phosphoric acid, and absorbance was measured at 450 nm. In contrast to the original dual-wavelength (450–620 nm) reading, our ELISA reader performs automatic baseline correction at 450 nm with consistent blank subtraction; this approach provides equivalent background correction and does not affect relative signal discrimination. The assay was validated using a panel of controls, including mouse anti-NP monoclonal antibodies, CCHFV IgG-positive human sera, and naïve human sera. All samples were tested in duplicate, and the mean optical density (OD) values were used for analysis. The cutoff value for positivity was calculated as the mean OD of negative-control sera plus three standard deviations, consistent with the established protocol [13].

The relationship between seroreactivity and survey data was assessed via univariate chi-square analysis using an alpha of 0.05 in SAS 9.6 (Cary, NC).

## Results

**Fig 1:**
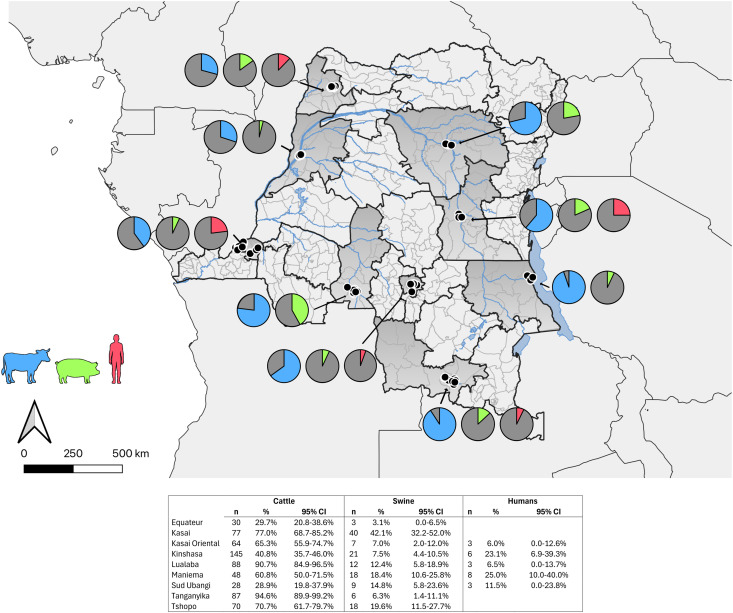
CCHFV seroreactivity among cattle, swine, and agriculture workers in the Democratic Republic of the Congo. Shapefile from https://data.humdata.org/dataset/dr-congo-health-0. DRC map base layers are provided by United Nations Office for the Coordination of Humanitarian Affairs [14].

Among the 1,118 cattle surveyed across nine provinces 57.0% (95%CI: 54.1-59.9%) were seroreactive. Cattle from two provinces in the southeast, Tanganyika and Lualaba, had 94.6% (95%CI: 89.9-99.2%) and 90.7% (95%CI: 84.9-96.5%) reactivity, respectively. Among the 1,020 swine surveyed 13.4% (95%CI: 11.1-15.2%) were seroreactive. Swine from Kasai province had 42.1% (95%CI: 32.2-52.0%) seroreactivity, the highest rate of seroreactivity among swine (Fig 1). Attached ticks were found on 592 (52.9%) of all cattle and four (0.4%) of swine at the time of collection; seroreactivity was not associated with the presence of ticks (Table 1). Among the collected ticks, 16 *Hyalomma rufipes* were identified on nine cattle from four provinces: Kinshasa (6), Sud-Ubangi (1), Kasai (1), and Tanganyika (8). Among these nine cattle, six were seroreactive for CCHFV. Very few (<1%) cattle and swine were experiencing illness or exhibiting symptoms at the time of data collection. Two other tick genera we identified: *Amblyomma* (n = 1401, 91.4%) and *Rhipicephalus* (n = 115, 7.5%).

**Table 1:** Livestock Seroreactivity.

	Cattle					Swine				
	All N = 1118		Seroreactive n = 637 (57.0%)			All N = 1020		Seroreactive n = 134 (13.4%)		
	N	%	N	%	Chi-sq p value	N	%	N	%	Chi-sq p value
**Province**										
Tanganyika	92	8.2	87	13.7	<.0001	96	9.4	6	4.5	<.0001
Lualaba	97	8.7	88	13.8		97	9.5	12	9.0	
Kasai	100	8.9	77	12.1		95	9.3	40	29.9	
Tshopo	99	8.9	70	11.0		92	9.0	18	13.4	
Kasai Oriental	98	8.8	64	10.1		100	9.8	7	5.2	
Maniema	79	7.1	48	7.5		99	9.7	18	13.4	
Kinshasa	355	31.8	145	22.8		282	27.6	21	15.7	
Equateur	101	9.0	30	4.7		98	9.6	3	2.2	
Sud Ubangi	97	8.7	28	4.4		61	6.0	9	6.7	
**Sex**										
Female	749	67.0	467	73.3	<.0001	665	65.2	103	76.9	0.0023
Male	369	33.0	170	26.7		355	34.8	31	23.1	
**Age**										
Infant	117	10.5	45	7.1	<.0001	31	3.0	4	3.0	0.0135
Juvenile	240	21.5	119	18.7		426	41.8	43	32.1	
Adult	745	66.6	465	73.0		561	55.0	86	64.2	
Senior	15	1.3	8	1.3		1	0.1	1	0.8	
Geriatric	1	0.1	0	0.0		1	0.1	0	0.0	
**Body Condition Score**										
2	16	1.4	5	0.8	<.0001	73	7.2	5	3.7	0.0144
3	298	26.7	148	23.2		514	50.4	58	43.3	
4	612	54.7	393	61.7		381	37.4	60	44.8	
5	154	13.8	73	11.5		48	4.7	9	6.7	
6	38	3.4	18	2.8		4	0.4	2	1.5	
**Lesions**										
No	1073	96.0	613	96.2	0.6143	975	95.6	127	94.8	0.6233
Yes	45	4.0	24	3.8		45	4.4	7	5.2	
**Ticks**										
No	526	47.1	298	46.8	0.8372	1016	99.6	134	100.0	0.4358
Yes	592	53.0	339	53.2		4	0.4	0	0.0	

Of the 180 agricultural workers surveyed, 12.8% (95%CI: 7.9-17.6%) (23) were seroreactive for CCHFV antibodies. Maniema and Kinshasa provinces had the greatest number of seroreactive individuals, 8 (25%, 95%CI: 10.0-40.0%) and 6 (26.1%, 95%CI: 6.9-39.3%), respectively. Among all participants, 65.6% reported that they had contact with a sick animal in the past year. While there was a slightly greater proportion of seroreactivity among those who had contact compared with those who did not (15.2% vs 8.5%), the relationship was not statistically significant (chi-sq p = 0.2060). The use of personal protective equipment (PPE) was limited as 80% reported that they rarely or never wear PPE. 55% of participants reported that they live on the farm property. Across the cohort, 16.1% reported that they never wash their hands during their workday, 35.6% reported handwashing once or twice daily. Outside of work, 86.1% of agricultural workers reported that they entered the forest, 34.4% reported hunting wild animals, and 53.3% reported eating bushmeat (Table 2).

**Table 2:** Farmer Demographics by Seroreactivity.

	All N = 180		Negative n = 157 (87.2)		Seroreactive n = 23 (12.8)		Chi-sq p value
	N	%	N	%	N	%	
**Province**							
Kasai Oriental	50	27.78	47	29.94	3	13.04	0.0331
Kinshasa	26	14.4	20	12.7	6	26.09	
Lualaba	46	25.56	43	27.39	3	13.04	
Maniema	32	17.78	24	15.29	8	34.78	
Sud Ubangi	26	14.44	23	14.65	3	13.04	
**Site Type**							0.991
Commercial farm	132	73.33	115	73.25	17	73.91	
Village farm	33	18.33	29	18.47	4	17.39	
Abbatoir	15	8.33	13	8.28	2	8.7	
**Age**							
18-24	40	22.22	37	23.57	3	13.04	0.0109
25-34	44	24.44	41	26.11	3	13.04	
35-49	52	28.89	47	29.94	5	21.74	
50-75	44	24.44	32	20.38	12	52.17	
**Sex**							
Female	36	20	33	21.02	3	13.04	0.3710
Male	144	80	124	78.98	20	86.96	
**Primary Residence at Farm**							
No	81	45	68	43.32	13	56.52	0.1707
Yes	99	55	89	56.69	10	43.48	
**Contact with sick animals in last 12 months**							
No	59	32.78	54	35.06	5	21.74	0.2060
Yes	118	65.56	100	64.94	18	78.26	
**PPE Frequency**							
Never	124	68.89	109	69.43	15	65.22	0.3596
Rarely	20	11.11	16	10.19	4	17.39	
Sometimes	11	6.11	10	6.37	1	4.35	
Often	6	3.33	4	2.55	2	8.7	
Always	19	10.56	18	11.46	1	4.35	
**Handwashing**							
Never	29	16.11	27	17.2	2	8.7	0.1005
1–2 times per day	64	35.56	58	36.94	6	26.09	
Few times a day (3–5)	26	14.44	19	12.1	7	30.43	
Many times a day	61	33.89	53	33.76	8	34.78	
**Have you ever done the following activities?**							
Enter a cave or mine	27	15	24	15.29	3	13.04	0.8898
Visit the forest	155	86.11	135	85.99	20	86.96	0.9001
Hunt wild animals	62	34.44	54	34.39	8	34.78	0.9708
Slaughter/ butcher wild animals	80	44.44	71	45.22	9	39.13	0.5829
Prepared wild animals to eat	96	53.33	82	52.23	14	60.87	0.4379

Of the 56 cattle farms visited, all but one farm in Kinshasa province had at least one seroreactive animal. On average, 59.5% of cattle tested at each facility were seroreactive to CCHFV; at seven farms, three in Lualaba province and four in Tanganyika, all animals tested were seroreactive. Among the 65 swine farms visited, 56.9% (37) had at least one seroreactive animal on the day of sampling. On average, we observed 21.5% seroreactivity among selected swine among farms that had at least one seroreactive animal.

All but three (94.5%) cattle farmers reported that they allow their cattle to graze and 87.5% reported that they had an issue with ticks within their herd. Among swine herds, 16.9% allow grazing and 3 farms (4.6%) reported tick problems. During collection it was observed that pigs were often kept in small, wet enclosures unsuitable for ticks compared with grassier and more open areas where cattle were kept. For both cattle and swine, the use of vaccines was less common, 19.6% and 21.5% of farms, respectively; however the use of any kind of veterinary medicine was very common—73.2% of cattle farms and 67.7% of swine farms regularly used and had access to medicines. When asked about nose-to-nose contact with other species, rats (37.5%), mice (28.6%), and poultry (28.6%) were most common among cattle herds. Among swine herds, 55.4% of farmers reported nose-to-nose contact with rats and mice. Swine farms with at least one seroreactive animal reported much higher rates of contact with rodents compared to those that did not— for rats: 66.7% vs 33.3%, Chi-sq p = 0.0314; for mice: 69.4% vs 30.6%, Chi sq p = 0.0083 (Table 3).

**Table 3:** Farm Management Practices.

	Cattle Farms N = 56		Swine Farms N = 65	
	N	%	N	%
**Herd size**				
one-10	3	5.36	7	10.77
ten-25	10	17.86	39	60
25-50	21	37.5	13	20
50-100	13	23.21	5	7.69
100-250	8	14.29	1	1.54
**History of reporting animal diseases**				
No	37	66.07	48	73.85
Yes	19	33.93	17	26.15
**Do you allow your animals to graze?**				
No	3	5.36	54	83.08
Yes	53	94.64	11	16.92
Familiarity with common livestock diseases*				
*missing*	20	35.71	28	43.08
Never heard of it	6	10.71	12	18.46
Recognized some from the list	7	12.5	4	6.15
I know some basics	9	16.07	13	20
I am well informed	14	25	8	12.31
**Are there any tick problems in your herd?**				
No	7	12.5	62	95.38
Yes	49	87.5	3	4.62
**Do you routinely vaccinate your herd?**				
No	44	78.57	51	78.46
Yes	11	19.64	14	21.54
**Do you regularly use medicines?**				
No	15	26.79	21	32.31
Yes	41	73.21	44	67.69
**Nose-to-nose contact in the last 12 months**				
Bat	8	14.29	8	12.31
Rat	21	37.5	36	55.38
Mouse	16	28.57	36	55.38
Dog	11	19.64	13	20
Cat	4	7.14	12	18.46
Poultry	16	28.57	14	21.54
Monkey	0	0	0	0

*Cattle diseases listed: Johne’s disease, viral diarrhea, brucellosis, tuberculosis, mastitis, mycoplsma, pneumonia, babesiosis, anaplasmosis, foot and mouth disease, anthrax, Contagious bovine pleuropneumonia, rift valley fever, bluetongue, otitis.

Swine diseases listed: Salmonellosis, African swine fever, streptococcus typanomosis, colibacillosis, swine erysipelas, coccidosis, diarrhea.

## Discussion

This survey represents the largest integrated One Health CCHFV serologic survey in the DRC to date. In this paper, we focus on a defined cohort of agricultural workers with direct livestock contact, paired with concurrent sampling of cattle and swine—the first reported CCHFV seroprevalence data for swine in the DRC. In addition to serology, we collected and identified attached ticks, enabling integration of vector ecology with host infection status. Detailed farm management and biosecurity practices, along with participant behavioral data (PPE use, hygiene practices, wildlife contact), allowed us to identify modifiable on-farm and individual risk factors. Together, these features provide a high-resolution, One Health perspective on CCHF disease risk in DRC.

The DRC has two seasons—dry and rainy. Due to logistical constraints and the size of this serologic study, Equateur, Kasai, Lualaba, Maniema, Sub Ubangi, and some Kinshasa sites were visited during the rainy season; Kasai Oriental, Tanganyika, Tshopo, and some Kinshasa sites were visited during the dry season. Previous work in sub-Saharan Africa and Iran have demonstrated that precipitation has a negative association with tick abundance and may impact CCHFV prevalence in the environment [15,16]. While Lualaba was visited during the rainy season and Tanganyika during the dry, CCHFV seroreactivity among cattle and swine in this southeastern region was quite high, nearly ubiquitous on some farms. This may be due to the savannah-mosaic grasslands which provide a more suitable habitat for ticks in comparison with forested regions of the country. Spatial analysis suggests that the risk of CCHF disease occurrence is higher in arid and semi-arid lands [15]. Indeed, in Uganda seroprevalence surveys of CCHFV in livestock have found rates of seroreactivity to be very high (~90%) [9,17]. In Tanzania, which neighbors Tanganyika, fewer seroprevalence studies have been conducted but a 2016 study found 50% seropositivity in cattle [18]. Further models on CCHFV serosurveillance and environmental factors are needed to explore this spatial relationship.

While the observed seroreactivity indicates a high potential for CCHFV transmission among livestock and humans, only 16 *Hyalomma rufipes* were identified from ticks found on nine cattle. While *Hyalomma* ticks are considered the primary vector for CCHFV, the other two tick genera we identified *Amblyomma* (n = 1401, 91.4%) and *Rhipicephalus* (n = 115, 7.5%) can also transmit the virus, and these species were found in abundance [19–21]. Detection of the presence of CCHFV among collected ticks from seroreactive livestock can provide further understandings of CCHFV carriage among tick genera. Indeed, 87.5% of farms reported tick infestations among their herds. All collected attached ticks were homogenized, and following RNA extraction (QIAGEN), tested for CCHFV with commercially available RT-PCR kits. All measured cycle threshold (Ct) values exceeded established validated assay cut offs and thus were considered negative for CCHFV.

We observed a statistically significant relationship between swine farms with a seroreactive animal and the rate of nose-to-nose contact between the herd and rodents. On cattle farms this relationship was not significant but around a third of farms reported rodent contact. While rodents are not considered a primary reservoir for CCHFV they may play an indirect role in the transmission chain as a host for the tick vector, an amplifier, or bridge host between questing ticks and livestock [22,23].

Interestingly, we did not observe a pattern between human and cattle seroprevalence. In Lualaba, where 90.7% of cattle were seroreactive, just 6.5% of samples agricultural workers were seroreactive. Further scrutiny is needed to understand the causal pathway of CCHFV infection in agricultural workers and the role that livestock play as intermediate vectors. In general, we did observe that the agricultural workers surveyed likely experience frequent low-level exposures during their job duties and lifestyles outside of work that have an additive effect over their lifetimes. Handwashing and PPE use were limited, and a majority of individuals interacted closely with sick animals. We observed a greater proportion of seropositivity among those aged 50 years or older compared with younger age groups which may indicate that there is repeated CCHFV exposure and risk across the lifecourse. Seroreactivity was not associated with any reported zoonotic exposures: visiting the forest, hunting wild animals, slaughtering or butchering wild animals, or preparing wild animals to eat. However, 80% of participants reported that they entered the forest regularly for personal reasons, indicating that environmental and zoonotic exposures are commonplace across the DRC. Further laboratory work to detect active or recent infection is needed to further understand the risks of CCHFV transmission in agricultural environments.

Two prior DRC serosurveys reported lower—or highly variable—anti-CCHFV IgG seropositivity. In 2013, a ruminant survey in southeastern DRC detected 2/514 seropositive cattle (≈0.4%) [24]. A subsequent analysis of archived sera collected in 2017–2019 from humans and cattle, tested with the same in-house NP-ELISA used here, found 4.4% seropositivity in humans and 42.8% in cattle [25]. Differences between those estimates and ours likely reflect geography, sampling frame, host species, exposure risks, timing/seasonality, and assay performance. Longitudinal studies, coupled with entomological sampling, are needed to clarify trends in livestock infection and tick infection rates. Assay choice is also pivotal: performance varies across platforms—for example, a 2017 Ugandan study reported 12.6% seropositivity using an in-house assay versus 75% with a commercial IDVet ELISA [26]. While the in-house assay used here performed well compared to a widely used commercial kit, harmonized assays and standardized cut-offs will be essential for future CCHFV serosurveillance. Additionally, while the assay primarily detects IgG, it may also detect other immunoglobulin isotypes with affinity for Protein A/G in some species.

Overall, this One Health cross-sectional survey found the exposure to CCHFV over the life course of cattle to be greater than anticipated and reinforces WHO’s designation of CCHF disease as a priority pathogen for endemic preparedness. This survey was cross-sectional, and as such represents only a snapshot of potential CCHF disease risk in the DRC. Further longitudinal research can provide important understandings of CCHF disease risk over time. As only 16 *Hyalomma* ticks were identified, targeted research of questing ticks, rather than feeding ticks could provide important data on tick habitat in the DRC. Additional testing on collected ticks could provide further understandings of CCHFV transmission in agricultural settings.

The DRC occupies a strategically important position for zoonotic surveillance. It is home to a high density of human-livestock interaction, emerging land use change, climate-sensitive vector habitats, and limited healthcare access—all contributing factors to pathogen spillover risk. Agricultural workers are at heightened risk for zoonotic transmission and proper health reporting systems and communication regarding occupational safety should be prioritized to support CCHFV control and outbreak preparedness.

## Supporting information

S1 Data Survey data. (CSV)
